# Factors That Mattered in Helping Travelers From Countries With Ebola Outbreaks Participate in Post-Arrival Monitoring During the 2014-2016 Ebola Epidemic

**DOI:** 10.1177/0046958019894795

**Published:** 2019-12-18

**Authors:** Christine E. Prue, Peyton N. Williams, Heather A. Joseph, Mihaela Johnson, Abbey E. Wojno, Brittany A. Zulkiewicz, John Macom, Jennifer P. Alexander, Sarah E. Ray, Brian G. Southwell

**Affiliations:** 1 Centers for Disease Control and Prevention, Atlanta, GA, USA; 2 RTI International, Research Triangle Park, NC, USA

**Keywords:** Ebola virus disease, travel, health promotion, trust, monitoring

## Abstract

During the 2014-2016 Ebola epidemic in West Africa, the US Centers for Disease Control and Prevention (CDC) developed the CARE+ program to help travelers arriving to the United States from countries with Ebola outbreaks to meet US government requirements of post-arrival monitoring. We assessed 2 outcomes: (1) factors associated with travelers’ intention to monitor themselves and report to local or state public health authority (PHA) and (2) factors associated with self-reported adherence to post-arrival monitoring and reporting requirements. We conducted 1195 intercept in-person interviews with travelers arriving from countries with Ebola outbreaks at 2 airports between April and June 2015. In addition, 654 (54.7%) of these travelers participated in a telephone interview 3 to 5 days after intercept, and 319 (26.7%) participated in a second telephone interview 2 days before the end of their post-arrival monitoring. We used regression modeling to examine variance in the 2 outcomes due to 4 types of factors: (1) programmatic, (2) perceptual, (3) demographic, and (4) travel-related factors. Factors associated with the intention to adhere to requirements included clarity of the purpose of screening (*B* = 0.051, 95% confidence interval [CI], 0.011-0.092), perceived approval of others (*B* = 0.103, 95% CI, 0.058-0.148), perceived seriousness of Ebola (*B* = 0.054, 95% CI, 0.031-0.077), confidence in one’s ability to perform behaviors (*B* = 0.250, 95% CI, 0.193-0.306), ease of following instructions (*B* = 0.053, 95% CI, 0.010-0.097), and trust in CARE Ambassador (*B* = 0.056, 95% CI, 0.009-0.103). Respondents’ perception of the seriousness of Ebola was the single factor associated with adherence to requirements (odds ratio [OR] = 0.81, 95% CI, 0.673-0.980, for non-adherent vs adherent participants and OR = 0.86, 95% CI, 0.745-0.997, for lost to follow-up vs adherent participants). Results from this assessment can guide public health officials in future outbreaks by identifying factors that may affect adherence to public health programs designed to prevent the spread of epidemics.

**What do we already know about this topic?**
Many factors influence adherence to behavioral recommendations including knowledge, motivations, intentions, social norms, availability of tools needed to perform behaviors, message source credibility, and trust in government.
**How does your research contribute to the field?**
Predictors of intention or behavior included perceptions of threat, trust in government, family support for performing behaviors, ease of following instructions, and confidence in performing behaviors.
**What are your research’s implications toward theory, practice, or policy?**
Education efforts should ensure people understand the purpose of health interventions, know how to adhere to requirements, are equipped with needed tools, and trust program officials.

## Introduction

During the 2014-2016 Ebola epidemic in West Africa, novel approaches were developed to assess and manage the risk of travelers arriving to the United States from countries with Ebola outbreaks.

### Enhanced Entry Risk Assessment and Post-Arrival Monitoring

In October 2014, after 2 imported cases and an associated contact investigation of Ebola in the United States,^1-3^ US Centers for Disease Control and Prevention (CDC) revised previously issued movement and monitoring guidance to recommend active monitoring (where travelers had to take their temperature and evaluate themselves for Ebola symptoms twice a day and communicate at least once a day with a state or local public health authority [PHA]), and direct active monitoring (where public health workers had to make a direct contact with the traveler at least once a day to see if they have fever or other Ebola symptoms) in some circumstances, of travelers arriving from countries with Ebola outbreaks.^4^ Based on this guidance, travelers at designated US ports of entry were to undergo an enhanced risk assessment which classified them as having “low but not zero risk,” “some risk,” and “high risk.” Those who were designated as “low but not zero risk” were recommended to be actively monitored for 21 days after the last potential Ebola virus exposure.

US Customs and Border Protections (CBP) and CDC partnered at 5 US ports of entry to conduct the enhanced entry risk assessment at Chicago O’Hare International Airport (ORD), Hartsfield-Jackson Atlanta International Airport (ATL), Newark Liberty International Airport (EWK), John F Kennedy International Airport (JFK), and Washington Dulles International Airport (IAD).^5^ All air travelers who had been in countries with Ebola outbreaks were directed through these airports. Upon arrival, the travelers were directed to a screening area where the risk assessment was conducted. The risk assessment involved asking travelers 5 questions, observing for and asking about symptoms, and taking their temperature. US Customs and Border Protections officers collected travelers’ destination and contact information, which CDC passed to the receiving PHA.^4,5^ For most travelers, CBP assigned a risk level of “low but not zero.”

### CARE Kit

To help travelers self-monitor and communicate with the state or local PHA, CDC created the Check and Report Ebola Kit (CARE Kit), consisting of a digital thermometer, a symptom and temperature log, graphical depictions of Ebola symptoms, contact information for PHAs by jurisdiction, and a wallet-sized CARE card that reminded travelers to monitor their health, contained instructions for safely seeking care if needed, and alerted health care workers of possible Ebola exposure.^6^ US Customs and Border Protections officers gave the CARE Kit to travelers after they completed the risk assessment.

### Launch of CARE+

State and local PHAs struggled to monitor all travelers consistently in the initial weeks of the program.^7,8^ In response, approximately 6 weeks after CDC released its recommendations for post-arrival monitoring, CDC launched the CARE+ program, which introduced CARE Ambassadors.^8,9^ CARE Ambassadors were health educators trained to explain monitoring requirements and teach travelers how to use CARE Kit tools. They met with travelers for 5 to 8 minutes after CBP finished the risk assessment process.^9^ Ambassadors also gave travelers a cellular flip phone with at least 21 days of unlimited voice and text service and showed travelers how to use the phone. The CARE phone number was also provided to the state or local PHA to facilitate initial contact and continued communication between the traveler and the PHA.

CARE+ was developed in response to the observed challenges with program implementation and with behavioral science principles, which suggest that adherence to behavioral recommendations reflects not only people’s knowledge, motivations, and intentions but also message source credibility, social norms, and availability of resources or tools needed to perform recommended behaviors.^10-15^ People appear more likely to share personal information when they believe those requesting the information are trustworthy.^16-20^

### CARE+ Evaluation

The assessment of the CARE+ program included factors associated with intentions to adhere and with self-reported adherence to requirements. Our study does not speak to the value of screening as a public health strategy, per se. Several behavioral and information processing theories guided this assessment.^10-15,21-23^ We aimed to answer this question: What were the programmatic and perceptual predictors of travelers’ intentions to adhere to post-arrival monitoring and reporting requirements?

## Methods

We collected information in 3 phases: an in-person intercept interview at the airport, a first telephone interview, and a second telephone interview. In all phases, interviewers were trained and supervised by a project staff member.

### Airport Intercept Interviews

From April through June 2015, we conducted airport intercept interviews at JFK and Dulles airports with travelers arriving from countries with Ebola outbreaks who were 18 years or older and spoke either English or French. John F Kennedy International Airport and Dulles airports received the heaviest volume of travelers from countries with Ebola outbreaks. We conducted the interviews during times when traveler volume from Guinea, Liberia, or Sierra Leone was highest.

We approached 2426 travelers at the airport immediately after the travelers’ encounter with a CARE Ambassador, and 1195 travelers (49.3%) agreed to and completed the airport interview. Of the 1231 who did not complete the airport interview, 692 (56.2%) refused, 225 (18.3%) spoke a language other than English or French, 112 (9.7%) were under age, and 202 (16.4%) could not finish the interview (eg, the traveler terminated early to catch a flight).

The airport interview lasted about 10 minutes with interviewers recording responses on handheld electronic tablets. After the conclusion of the interview, we asked participants if they would be willing to take part in a telephone interview and, if they agreed, we asked for a phone number to reach them.

### Telephone Interviews

During the airport interview, 1041 travelers agreed to participate in a telephone interview with 654 (62.8%) completing this interview. Those who consented to the telephone interview were called within 5 days of their airport interview. Of the 387 who agreed to participate in a telephone interview but did not, 316 (81.7%) could not be contacted (eg, the phone number they provided us did not reach them), 69 (17.8%) refused, and 2 (0.01%) terminated early.

During the first telephone interview, 562 travelers agreed to participate in a second telephone interview, and of these, 319 (56.8%) completed the interview which was conducted 2 days before the end date of the traveler’s monitoring period. Of the 243 who agreed to participate in a second telephone interview but did not, 213 (87.7%) could not be contacted, 28 (11.5%) refused, and 2 (0.01%) terminated early. Computer-assisted telephone interviewing systems were used for telephone interviews which ended in July 2015.

Of our total sample of 1195, 541 participants were interviewed only at the airport and we could not reach them for a telephone interview. We designated these as “lost to follow-up.”

The CDC determined this assessment to be non-research, evaluation of public health response activities, and the US Office of Management and Budget approved data collection (OMB Control No. 0920-0932).

### Measures

The 3 phases of interviews consisted of questions about the traveler’s experience with the CARE+ program and factors that could influence their intention and ability to meet requirements (Tables 1 and 2). Measures were based on questions with yes/no response options, Likert scales, multiple-choice responses, open-ended items, and indices created from responses from multiple questions.

**Table 1.** Description of Independent Variables Used to Assess the US Centers for Disease Control and Prevention’s CARE+ Program During the 2014-2016 Ebola Epidemic.

Measures	Interview question and response options		Interview phase		
Concept or theory-based construct	Interview question	Response options	Airport interview	First telephone interview	Second telephone interview
Trust in CARE Ambassadors^a^	I (still) trust the CARE Ambassador as a source of information about Ebola.	Likert scale from 1 (strongly disagree) to 5 (strongly agree)	X	X (still trust)	
	The CARE Ambassador was knowledgeable as a source of information about Ebola.	Likert scale from 1 (strongly disagree) to 5 (strongly agree)	X	X	
	I (still) have confidence in the CARE Ambassador as a source of information about Ebola.	Likert scale from 1 (strongly disagree) to 5 (strongly agree)	X	X (still have confidence)	
	The CARE Ambassador is (was) a credible source of information about Ebola.	Likert scale from 1 (strongly disagree) to 5 (strongly agree)	X	X (was)	
	The CARE Ambassador who talked with me about the actions I must take cares (cared) about me as a person.	Likert scale from 1 (strongly disagree) to 5 (strongly agree)	X	X (cared)	
Trust in PHA^a^	I trust the people at the health department as a source of information about Ebola.	Likert scale from 1 (strongly disagree) to 5 (strongly agree)		X	X
	The people at the health department are knowledgeable as a source of information about Ebola.	Likert scale from 1 (strongly disagree) to 5 (strongly agree)		X	X
	I have confidence in the people at the health department as a source of information about Ebola.	Likert scale from 1 (strongly disagree) to 5 (strongly agree)		X	X
	The people at the health department care about me as a person	Likert scale from 1 (strongly disagree) to 5 (strongly agree)		X	X
Clarity of screening purpose	How clear was the purpose of Ebola screening?	1 Very unclear 2 Somewhat unclear 3 Clear 4 Very clear	X		
Ease of following CARE Kit instructions	How easy or difficult will it be for you to follow the instructions in the CARE Kit in the next few weeks?	1 Very difficult 2 Difficult 3 Easy 4 Very easy	X		
	How easy or difficult has it been for you to do these things?	1 Very difficult 2 Difficult 3 Easy 4 Very easy		X	
Confidence to check symptoms	How confident are you that you can check yourself for the next few weeks for symptoms of Ebola?	1 Not confident at all 2 Somewhat unconfident 3 Confident 4 Very confident	X	X	
Knowledge of program requirements	Based on what you’ve heard so far, how long do you need to do health checks for Ebola?	1 month 21 days 1 week Unsure	X		
Perceived seriousness of Ebola (personally)	How serious of a health concern is Ebola to you personally?	1 Not serious at all 2 Somewhat serious 3 Serious 4 Very serious	X	X	X
Perceived seriousness of Ebola (globally)	How serious of a health concern is Ebola to the world?	1 Not serious at all 2 Somewhat serious 3 Serious 4 Very serious	X	X	X
Likelihood of getting sick	In your opinion, how likely do you think it is that you will get sick with Ebola?	1 Very unlikely 2 Somewhat unlikely 3 Likely 4 Very likely	X	X	X
Approval of others for monitoring	People who are important to me, like friends or family, will approve of me checking myself for Ebola.	Likert scale from 1 (strongly disagree) to 5 (strongly agree)	X	X	X
Perceived caring of the CARE Kit creators	The people who wrote the CARE Kit materials care about me as a person.	Likert scale from 1 (strongly disagree) to 5 (strongly agree)	X	X	X
Having a non-CARE+ thermometer	Other than the thermometer you just received in your CARE Kit, do you have a thermometer where you are going to be staying?	0 No 1 Yes	X		
Having a non-CARE+ cell phone	Other than the CARE cell phone you just received during this process, do you have a cell phone that works in the United States?	0 No 1 Yes	X		

*Note.* CARE+ = Check and Report Ebola; PHA = public health authority.

a The scales for trust in Ambassadors and trust in the PHA, using 5 items each, were assessed for internal consistency using Cronbach’s alpha. We found high consistency. For the Ambassador trust scales, the alpha was .903 and .884 at airport interview and first telephone interview, respectively; for trust in PHA, .928 and .893 at first telephone interview and second telephone interview, respectively.

**Table 2.** Description of Dependent Variables Used to Assess the US Centers for Disease Control and Prevention’s CARE+ Program During the 2014-2016 Ebola Epidemic.

Measures	Interview question and response options		Interview phase		
Concept or theory-based construct	Interview question	Response options	Airport interview	First telephone interview	Second telephone interview
Intention to adhere to monitoring and reporting requirements	How likely is it that you will report temperature and symptoms to the health department every day for the next few weeks?	1 Very unlikely 2 Somewhat unlikely 3 Likely 4 Very likely	X	X	
	If you have a temperature of 100°F or 38°C, how likely would you be to seek medical care?	1 Very unlikely 2 Somewhat unlikely 3 Likely 4 Very likely	X	X	
Self-reported fulfillment of reporting requirements (adherence index)	Did you check your temperature twice yesterday?	0 No 1 Yes		X	X
	Yesterday did you check yourself for any other symptoms that were mentioned in the CARE Kit?	0 No 1 Yes		X	X
	Yesterday did you record or write down your temperature and any symptoms mentioned in the CARE Kit?	0 No 1 Yes		X	X
	Did you report your temperature and symptoms to the health department yesterday?	0 No 1 Yes		X	X

*Note.* CARE+ = Check and Report Ebola.

### Independent Variables

Independent variables included the traveler’s trust in the CARE Ambassadors and PHAs, knowledge and beliefs about Ebola, knowledge of requirements, perceptions of program attributes, beliefs about ease or difficulty in meeting requirements, and supports for fulfilling requirements (Table 1).

### Dependent Variables

Dependent variables included (1) the traveler’s stated intention to meet post-arrival monitoring and reporting requirements; that is, what do the travelers say they will do, and (2) self-reported fulfillment of post-arrival monitoring and reporting requirements; that is, what did the travelers say they actually did (Table 2).

The post-arrival monitoring and reporting requirements (hereafter “requirements”) were the CDC requirements for all travelers to (1) check their temperature twice a day, (2) check themselves for symptoms, (3) record their temperature and symptoms, and (4) report to the PHA each day.

We created an “adherence index” of the 4 self-reported behaviors. If travelers reported that they conducted all 4 behaviors, they were coded as “adherent” to requirements; otherwise they were coded as “non-adherent.” Only travelers who completed at least the first telephone interview and answered all 4 adherence questions could be classified for adherence.

### Covariates

Covariates included a travelers’ arrival airport (JFK or Dulles), if they “work in the field of public health or health care” (“yes” or “no”), if that day was the first time they had gone through an Ebola screening process in a US airport (“yes” or “no”), and date of arrival. For date of arrival, we dichotomized if (1) the person arrived on or before May 9, 2015, or (2) they arrived after May 9, since Liberia was first declared free of Ebola virus transmission on May 9. This time factor was examined because this declaration may have affected travelers’ beliefs about their need to fulfill post-arrival monitoring requirements in the United States.

Finally, we retrospectively pulled demographics from the Quarantine Activity Reporting System (QARS), a CDC system that records demographic and other data from travelers arriving in the United States.^24^ For each participant, CDC pulled the passport country (from what country or countries did the traveler hold a passport), the country or countries with an Ebola outbreak that the traveler had been in, their sex, age, and the unique ID number on the CARE Card issued to the participant. For each traveler, we pulled the age as a continuous variable for ages 25 to 59. Because of the smaller number of younger and older travelers, other ages were put into categories for privacy reasons as follows: 18 to 24, 60 to 64, 65 to 69, and 70 and older. We linked the QARS data set with our data set via the CARE Cared ID number, which participants provided during the airport interview.

### Data Analysis

We used predictive regression models to account for variance in the dependent variables because we aimed to determine the effect of a series of independent variables (ie, predictors) on a dependent variable (ie, outcome). In this assessment, we used an ordinary least squared (OLS) regression model (which assumes a continuous dependent variable) to examine the effect of independent variables on adherence intentions. We used a multinomial logistic regression model (which assumes unordered categorical dependent variable) to assess the effect of independent variables on the odds of being in 1 of 3 groups (adherent, non-adherent, or lost to follow-up) based on the adherence index. We modeled the adherent group as the reference group, focusing on affirmed adherence as the key outcome in question. We reported the regression coefficient *B*, which in the case of OLS regression reflects the amount of change in the outcome that would be predicted by a unit change in the predictor and in the case of multinomial logistic regression reflects the change in the logit of the outcome relative to the referent group (ie, adherent group) based on a unit change in the predictor variable. We judged statistical significance based on a *P*-value less than .05. We used SAS Enterprise Guide (version 7, SAS Institute, Inc., Cary, NC, USA) for all analyses.

## Findings

### Participant Characteristics

Of the 1195 participants who completed the airport interview, almost all (99.1%) were conducted in English (Table 3). Nearly two-thirds of respondents were men (61.4%), and the average age was 42.9. John F Kennedy International Airport arrivals composed the larger portion of the sample (61.9%), and 21.9% of the sample reported working in public health or health care. Using the adherence index, 406 (34.0%) of the sample was adherent, 203 (17.0%) was non-adherent, 541 (45.3%) were lost to follow-up (eg, they did not complete the first telephone interview) and 45 (3.8%) could not be classified because they refused to answer 1 or more adherence questions. For most participants (85.9%), this entry constituted their first experience with the risk assessment process. Liberia was the country most frequently reported as the country of potential Ebola exposure (47.8%). US passport holders constituted the largest percentage of the sample (40.1%), followed by Liberia (29.1%), Sierra Leone (15.9%), Guinea (4.7%), and other countries (10.2%).

**Table 3.** Characteristics of Participants in an Evaluation of the US Centers for Disease Control and Prevention’s CARE+ Program During the 2014-2016 Ebola Epidemic.

	All participant (n = 1195) No. (%)	Airport interview only (n = 541) No. (%)	Airport interview and first telephone interview^a^ (n = 654) No. (%)	Completed interviews in all 3 phases (n = 319) No. (%)
Language of interview (English)	1184 (99.1)	537 (99.3)	647 (98.9)	315 (98.8)
Arrival airport				
JFK	740 (61.9)	320 (59.2)	420 (64.2)	223 (69.9)
IAD	455 (38.1)	221 (40.9)	234 (35.8)	96 (30.1)
First US Ebola entry risk assessment	1027 (85.9)	454 (83.9)	573 (87.6)	292 (91.5)
Country of potential exposure^b^				
Liberia	546 (46.2)	219 (41.2)	327 (50.2)	179 (56.3)
Guinea	212 (17.9)	117 (22.0)	95 (14.6)	34 (10.7)
Sierra Leone	403 (34.1)	182 (34.2)	221 (34.0)	102 (32.1)
Multiple countries	22 (1.9)	14 (2.6)	8 (1.2)	3 (0.9)
Sex—Male^b^	726 (61.4)	307 (57.7)	419 (64.4)	199 (62.6)
Age (mean, SD)^b^	42.9 (12.5)	42.5 (12.6)	43.2 (12.4)	43.8 (13.1)
Passport country^c^				
Liberia	344 (29.1)	118 (22.2)	226 (34.8)	150 (47.2)
Sierra Leone	188 (15.9)	70 (13.2)	118 (18.2)	58 (18.2)
Guinea	56 (4.7)	29 (5.5)	27 (4.2)	12 (3.8)
United States	474 (40.1)	248 (46.6)	226 (34.8)	82 (25.8)
Other	120 (10.2)	67 (12.6)	53 (8.2)	16 (5.0)
Work in public health or health care—Yes^d^	262 (21.9)	120 (22.2)	142 (21.7)	61 (19.1)
Had a non-CARE+ thermometer—Yes	580 (49.4)	281 (52.8)	299 (46.5)	130 (41.5)
Had a non-CARE+ cell phone—Yes	762 (63.8)	381 (70.6)	381 (58.3)	168 (52.7)

*Note.* CARE+ = Check and Report Ebola; JFK = John F Kennedy International Airport; IAD = Washington Dulles International Airport.

a Includes participants who completed all 3 interviews and those who completed only airport interview and first telephone interview.

b Sex, age, and exposure country were not available for 12 respondents.

c Passport country was not available for 13 respondents.

d Health care worker status was not available for 1 respondent.

### Regression Results

The results from an analysis of bivariate relationships and of the adjusted regression models predicting intentions to adhere showed several variables with positive and statistically significant relationships with intentions to adhere: trust in the CARE Ambassador (*B* = 0.056, 95% confidence interval [CI], 0.009-0.103), clarity of screening purpose (*B* = 0.051, 95% CI, 0.011-0.092), ease of following instructions (*B* = 0.053, 95% CI, 0.010-0.097), confidence in checking symptoms (*B* = 0.250, 95% CI, 0.193-0.306), seriousness of Ebola (*B* = 0.055, 95% CI, 0.031-0.077), and approval of others to perform post-arrival monitoring and reporting behaviors (*B* = 0.103, 95% CI, 0.058-0.148; Tables 4 and 5). These estimates represent the average increase in intention to adhere for 1 unit increase in the predictor. These predictors accounted for 18% of the variance in intentions.

**Table 4.** Bivariate Relationships Between Predictors and Intention to Adhere and Self-Reported Adherence to Monitoring and Reporting Requirements During an Evaluation of the US Centers for Disease Control and Prevention’s CARE+ Program During the 2014-2016 Ebola Epidemic.

Predictor	Bivariate relationships predicting *intention* to adhere with monitoring and reporting required actions		ORs from bivariate multinomial logistic regressions predicting *behavioral adherence*^a^ to monitoring and reporting required actions			
	*B*	95% CI	Non-adherent vs adherent		Lost to follow-up vs adherent	
			OR	95% CI	OR	95% CI
Attitudes and knowledge about CARE+						
Trust in CARE Ambassador^b^	0.174***	0.132-0.217	0.702*	0.506-0.975	0.566***	0.439-0.730
Clarity of screening^b^	0.124***	0.084-0.164	0.832	0.624-1.109	0.901	0.716-1.132
Ease of following instructions^b^	0.138***	0.095-0.180	0.966	0.705-1.324	0.821	0.647-1.042
Confidence in checking symptoms^b^	0.308***	0.255-0.360	0.913	0.621-1.342	1.054	0.777-1.431
Knowledge of monitoring duration^c^ (21 days vs all other responses)	−0.054	−0.141-0.034	1.032	0.563-1.891	1.246	0.772-2.011
Perceptions of threat about Ebola and approval from others to monitor and report						
Seriousness of Ebola personally^b^	0.062***	0.039-0.084	0.837*	0.706-0.993	0.759***	0.665-0.866
Likelihood of getting sick^b^	0.006	−0.044-0.057	1.349^†^	0.967-1.882	1.084	0.808-1.455
Approval from others^b^	0.180***	0.137-0.222	0.800	0.586-1.094	0.820	0.641-1.049
CARE+ program elements directly enabling monitoring and reporting behaviors						
Non-CARE+ thermometer^c^ (had vs did not have)	−0.003	−0.050-0.045	0.698*	0.494-0.985	1.177	0.908-1.525
Non-CARE+ cell phone^c^ (had vs did not have)	−0.017	−0.066-0.032	0.922	0.655-1.298	1.624***	1.238-2.130
Covariates						
Media coverage proxy^c,d^ (after vs before)	−0.015	−0.069-0.039	1.185	0.801-1.752	0.993	0.743-1.327
Airport ID^c^ (JFK vs IAD)	−0.013	−0.061-0.036	1.202	0.843-1.714	0.857	0.658-1.117
Health worker^c^ (health worker vs not health worker)	0.008	−0.048-0.065	0.838	0.552-1.270	0.975	0.716-1.328
First-time US Ebola entry risk assessment^c^ (first time vs not first time)	0.018	−0.049-0.086	0.726	0.446-1.184	0.683^†^	0.467-1.000
Passport country—West Africa	Ref^e^		Ref^e^		Ref^e^	
Passport country—United States	−0.014	−0.064-0.036	0.788	0.546-1.138	1.746***	1.323-2.303
Passport country—Other	−0.029	−0.110-0.052	0.668	0.341-1.310	1.967**	1.255-3.084
Country of potential exposure—Liberia	Ref^e^		Ref^e^		Ref^e^	
Country of potential exposure—Guinea	0.010	−0.056-0.075	0.847	0.516-1.390	1.629**	1.131-2.344
Country of potential exposure—Sierra Leone	−0.031	−0.084-0.023	0.750	0.514-1.095	1.068	0.798-1.430
Country of potential exposure—Multiple	0.057	−0.119-0.234	n/a^f^		1.726	0.682-4.366
Age^b^	0.001	−0.001-0.003	1.000	0.986-1.014	0.996	0.985-1.007
Sex^c^ (male vs female)	−0.083***	−0.131-–0.035	0.907	0.637-1.290	0.721*	0.552-0.942

*Note.* CARE+ = Check and Report Ebola; CI = confidence interval. OR = odds ratio; JFK = John F Kennedy International Airport; IAD = Washington Dulles International Airport.

a The dependent variable is completion of the telephone survey and reported previous-day adherence with 4 behaviors (reporting to health authorities, recording temperature, checking temperature, and checking for other symptoms) that make up an adherence index. Travelers who originally consented to be contacted but did not participate in the first telephone interview were coded as “lost to follow-up.”

b For Likert-scale predictors and age, the estimates represent the average increase in intention to adhere for 1 unit increase in the predictor. For instance, 0.174 represents the average increase in intention to adhere for 1 unit increase in trust in ambassador scale.

c For categorical predictors, the latter category in the table is the reference group. A positive estimate indicates that the non-reference group has higher intentions to adhere; a negative estimate indicates that the reference group has higher intentions to adhere. The estimate represents the mean difference in intention to adhere between the 2 groups.

d On May 9, 2015, Liberia was first declared free of Ebola virus transmission. Because this declaration may have affected travelers’ beliefs about their need to fulfill monitoring requirements in the United States, the association was examined.

e Reference group.

f Estimate is non-estimable because no one in non-adherent group listed multiple countries as potential exposure.

† *P* < .10. *P < .05. **P < .01. ***P < .001.

**Table 5.** Regression Models to Predict Intention to Adhere and Self-Reported Adherence to Monitoring and Reporting Requirements During an Evaluation of the US Centers for Disease Control and Prevention’s CARE+ Program During the 2014-2016 Ebola Epidemic.

Predictor	Adjusted estimates from linear regression model predicting *intention* to adhere with monitoring and reporting required actions		Adjusted odds ratios from multinomial logistic regression models predicting *behavioral adherence*^a^ to monitoring and reporting required actions			
	*B*	95% CI	Non-adherent vs adherent		Lost to follow-up vs adherent	
			OR	95% CI	OR	95% CI
Attitudes and knowledge about CARE+						
Trust in CARE Ambassador^b^	0.056*	0.009-0.103	0.797	0.536-1.184	0.615**	0.453-0.835
Clarity of screening^b^	0.051*	0.011-0.092	0.89	0.647-1.222	1.043	0.808-1.348
Ease of following instructions^b^	0.053*	0.010-0.097	1.018	0.716-1.447	0.879	0.670-1.152
Confidence in checking symptoms^b^	0.250***	0.193-0.306	1.081	0.694-1.683	1.243	0.878-1.761
Knowledge of monitoring duration^c^ (21 days vs all other responses)	0.006	−0.079-0.091	1.177	0.618-2.242	1.208	0.720-2.025
Perceptions of threat about Ebola and approval from others to monitor and report						
Seriousness of Ebola personally^b^	0.054***	0.031-0.077	0.812*	0.673-0.98	0.862*	0.745-0.997
Likelihood of getting sick^b^	0.006	−0.042-0.054	1.333	0.944-1.882	1.078	0.792-1.467
Approval from others^b^	0.103***	0.058-0.148	0.956	0.671-1.363	0.972	0.733-1.290
CARE+ program elements directly enabling monitoring and reporting behaviors						
Non-CARE+ thermometer^c^ (had vs did not have)	0.021	−0.029-0.070	0.68^†^	0.457-1.012	0.835	0.615-1.135
Non-CARE+ cell phone^c^ (had vs did not have)	−0.041	−0.094-0.011	1.151	0.768-1.727	1.446*	1.042-2.007
Covariates						
Media coverage proxy^c,d^ (after vs before)	−0.024	−0.075-0.026	1.229	0.814-1.856	0.988	0.724-1.349
Airport ID^c^ (JFK vs IAD)	−0.037	−0.086-0.012	1.078	0.726-1.601	0.948	0.702-1.279
Health worker^c^ (health worker vs not health worker)	0.003	−0.053-0.059	0.939	0.593-1.486	0.712^†^	0.503-1.009
First-time US Ebola entry risk assessment^c^ (first time vs not first time)	0.015	−0.050-0.081	0.669	0.396-1.129	0.841	0.557-1.272
Passport country—West Africa	Ref^e^		Ref^e^		Ref^e^	
Passport country—United States	−0.018	−0.075-0.039	0.838	0.531-1.324	1.535*	1.084-2.174
Passport country—Other	−0.024	−0.105-0.057	0.695	0.338-1.427	1.683*	1.026-2.761
Country of potential exposure—Liberia	Ref^e^		Ref^e^		Ref^e^	
Country of potential exposure—Guinea	−0.015	−0.079-0.049	0.821	0.481-1.404	1.390	0.935-2.066
Country of potential exposure—Sierra Leone	0.008	−0.044-0.060	0.788	0.523-1.188	1.038	0.754-1.430
Country of potential exposure—Multiple	0.035	−0.135-0.205	n/a^f^		1.210	0.431-3.401
Age^b^	0.0002	−0.002-0.002	1.001	0.987-1.017	0.994	0.982-1.005
Sex^c^ (male vs female)	−0.024	−0.070-0.023	0.957	0.658-1.391	0.694*	0.520-0.927

*Note.* For the regression examining intention to adhere, n = 1149, adjusted *R*^2^ = .18. For the regression examining adherence, n = 1106, Nagelkerke *R*^2^ = .10. CARE+ = Check and Report Ebola; CI = confidence interval. OR = odds ratio; JFK = John F Kennedy International Airport; IAD = Washington Dulles International Airport.

a The dependent variable is completion of the telephone survey and reported previous-day adherence with 4 behaviors (reporting to health authorities, recording temperature, checking temperature, and checking for other symptoms). Travelers who originally consented to be contacted but did not participate in the first telephone interview were coded as “lost to follow-up.”

b For Likert-scale predictors and age, the estimates represent the average increase in intention to adhere for 1 unit increase in the predictor (eg, for 1 unit increase in trust in ambassador, for 1 year increase in age), controlling for other variables in the model.

c For categorical predictors, the second category is the reference group. A positive estimate indicates that the non-reference group has higher intentions to adhere; a negative estimate indicates that the reference group has higher intentions to adhere. The estimate represents the mean difference in intention to adhere between the 2 groups, controlling for other variables in the model.

d On May 9, 2015, Liberia was first declared free of Ebola virus transmission. Because this declaration may have affected travelers’ beliefs about their need to fulfill monitoring requirements in the United States, the association was examined.

e Reference group.

f Estimate is non-estimable because no one in non-adherent group listed multiple countries as potential exposure.

† *P* < .10. *P < .05. **P < .01. ***P < .001.

Several predictors were statistically significant in the model predicting self-reported adherence to 4 required monitoring and reporting actions (Table 5). Specifically, perceptions of Ebola as serious resulted in 19% lower odds of being in the non-adherent group (OR = 0.812, 95% CI, 0.673-0.980) versus the adherent group. The impact of having a non-CARE thermometer on the odds of being in the non-adherent versus the adherent group was just above our threshold for significance.

Similarly, higher trust in CARE Ambassador or perceptions of Ebola as serious resulted in 38% (OR = 0.615, 95% CI, 0.453-0.835) and 14% (OR = 0.862, 95% CI, 0.745-0.997) lower odds, respectively, of being in the lost to follow-up group versus the adherent group. Having a non-CARE+ cell phone resulted in 45% (OR = 1.446, 95% CI, 1.042-2.007) higher odds of being in the lost to follow-up group compared with the adherent group.

Several covariates affected the odds of being in the lost to follow-up group versus the adherent group. Specifically, those who had a passport from the United States or a country other than a West African country had higher odds of being in the lost to follow-up group, and being a man was found to lower the odds of being in the lost to follow-up group. Being a health care worker resulted in lower odds of being in the lost to follow-up group, but this relationship was just above the significance threshold.

## Discussion

During the 2014-2016 Ebola epidemic, Farrar and Piot declared that “classic ‘outbreak control’ efforts are no longer sufficient for an epidemic of this size.” They went on to say that behavioral change interventions need to appreciate culture, be consensual, and be collaborative so that trust is built (or rebuilt).^25^ Population mobility, cultural norms, and a lack of trust in authority figures were noted as possible contributors to the epidemic.^25^ Sociologist Robert Dingwall noted, “The first line of defense will almost always be social and behavioral interventions that interrupt the movement of the disease through a population.”^26^ While there was not an Ebola outbreak in the United States nor were there any imported cases of Ebola after the monitoring program began, there was demand by the US public and lawmakers to take action. The post-arrival monitoring program was implemented as a less restrictive alternative to travel bans.^4^ Several studies have offered additional insights on important aspects of post-arrival monitoring programs for infectious diseases including: costs,^27^ reporting of false data,^28^ psychosocial impact and preferences for monitoring.^28^ Both articles point to the importance of applying risk communication principles and practices in outbreak responses.

The CARE+ program was developed to promote adherence to monitoring behaviors. Grounded in social and behavioral science, CARE+ included an interpersonal encounter in the preferred language of the traveler in which Ambassadors conveyed the key instructions for active monitoring, answered questions, provided tools needed to meet requirements, and demonstrated the use of those tools. The responses in this assessment substantiate the need for providing these tools: Half reported not having another thermometer, and more than a third reported not having another cell phone that worked in the United States. Tate et al’s survey of persons monitored by the New York City Department of Health and Mental Hygiene showed that respondents rated the prepaid cellular telephone they received as useful. In addition, more than twice as many respondents preferred conducting post-arrival monitoring over the telephone rather than via the Internet.^28^ The provision of thermometers and cell phones was important in the context of the expectation of 100% adherence to self-monitoring and reporting. Stehling-Ariza et al reported that less than 1% of post-arrival monitoring of 10 344 persons, done by 60 jurisdictions over a 5-month period, was incomplete and that almost 92% of persons monitored were travelers at low risk.^29^

Our results suggest that several factors mattered in helping travelers adhere to post-arrival requirements. Significant predictors of intention or behavior included an array of beliefs that future programs can address, including perceptions of threat, trust in government, family support for behaviors, ease of following instructions, and confidence in performing behaviors.

Trust in government can be an important influence on whether a person will adhere to government requirements. Messenger credibility is a well-established predictor of persuasion for attitude and behavior. The trustworthiness and knowledgeability dimensions of source credibility have varied influences.^30^ If the messenger is not credible, the message is more likely to be discounted and disregarded. Each of the West African countries had governmental responses to the outbreak that likely shaped perceptions of credibility or trust in the government. Those perceptions are also influenced by culture and history. The same is true with the US government and its citizenry. Trust is not static; actions can build or break trust over time.^31^ The CARE+ program was an attempt to personalize the US government in the form of a friendly person providing information and tools for travelers as they arrived in the United States. The CARE+ Ambassadors also set expectations that the travelers would be contacted by a local PHA who would give them additional information about the requirements for their jurisdiction, which varied.^32^ This handoff matters in creating a seamless system for implementing post-arrival monitoring, because many travelers may not know that public health functions in the United States are jurisdictional. Travelers with itineraries that took them to multiple jurisdictions would interact with multiple PHAs during their visit. Such situations could increase opportunities for travelers to be lost to follow-up or misunderstand varying requirements. Creators of government initiatives, especially those addressing emerging infectious diseases under intense public scrutiny, should consider how their programs or services build or break trust. In addition, they should consider the psychosocial effects a program may have on participants. Tate et al showed that many respondents experienced a range of feelings as a result of being monitored, such as annoyance, frustration, and stress.^28^ When asked what respondents found useful in helping to cope with being actively monitored, the top 2 reported answers were the public health post-arrival monitoring staff and support from family or friends.^28^

While trust between program implementers and participants appears to influence behavior, so does having the support of other people. Individuals act in accordance with norms and attitudes of those around them.^21^ Perceived support for reporting was associated with intentions to adhere to reporting requirements. Because taking the required actions could publicly signal potential exposure to Ebola, there were concerns that travelers might experience stigma. Tate et al’s survey showed that some respondents reported being treated differently by someone outside their household and by someone in their workplace.^28^ Acknowledgment of the importance of social support to combat stigmatization is likely a crucial step for various infectious diseases.

Most travelers reported that it was easy for them to follow the instructions provided. CARE Kit developers intended the instructions to be clear for an audience with low English literacy, offering step-by-step instructions in plain language and graphical depictions of required behaviors. Moreover, program staff were careful to ensure that the materials developed were culturally relevant for and respectful of travelers.^4^ Nevertheless, there were challenges in performing required behaviors.^4^ For some participants, using a digital thermometer or a flip phone may have been new—hence, the value of a CARE Ambassador who could tailor verbal instruction to a traveler’s need for information or skills.

Confidence to perform and having the tools to fulfill the required behaviors go hand in hand. Travelers expressed high confidence in their ability to perform behaviors, and findings showed that they had the tools needed. Some travelers preferred to use their own phones to report to PHAs, while others needed the CARE+ phone.^4^ Hennenfent et al’s assessment of travelers’ experiences with the District of Columbia’s post-arrival monitoring program showed that travelers perceived the program as beneficial and recommended that future programs distribute resources (eg, mobile phones) based on specific needs of the traveler.^8^ It should be noted that after local PHAs made initial contact, they informed travelers how to report in their jurisdiction. The varying reporting approaches included phone calls, Web-based reporting, and in-person visits or Skype interfaces that allowed the PHA to watch the travelers take their temperature.^4^ Reich et al offer a framework for assessing the cost-effectiveness of post-arrival monitoring and alternate strategies such as quarantine, pharmaceutical interventions, and risk communication.^27^

## Limitations

The findings in this report are subject to several important limitations. First, the assessment was based on a convenience sample which may introduce selection bias. While there were 5 airports to potentially sample from, the resources available for this assessment allowed the selection of only 2. There may have been differences in travelers between airports. We also could approach travelers only when interviewers were at the airport; travelers who arrived at off-hours could not be interviewed. Unfortunately, we have no way of knowing how our sample would vary from the larger universe of all travelers. Second, only 319 (26.7%) of our sample completed all 3 phases, so sample attrition from airport interview to the second telephone interview may have created fundamentally different groups. For example, US passport holders were the largest proportion of respondents who completed the airport interview (46.6%) but represent only 25.8% of those who completed all 3 phases. One possible reason for the attrition is that many respondents provided the interviewer with the CARE+ phone number but after establishing contact with the local PHA may have turned it off and used another phone. Some may have left the United States, while others may have lost interest in participating or felt overwhelmed by the required communications with PHAs. Third, the retrospective data linkage to QARS to associate demographic information to our participants could have introduced inaccuracy caused by potential QARS data entry error. Finally, all responses were self-reported and not verified so were subject to social desirability bias.

## Conclusions and Practical Implications

We present insights gleaned from an evaluation of CARE+, a program designed to support the post-arrival monitoring recommended by CDC to prevent the importation and transmission of Ebola virus in the United States during the 2014-2016 Ebola virus epidemic. This assessment identified predictors of intention and self-reported behavior that can be addressed by integrating social and behavioral science principles in the design of health interventions aimed at preventing the spread of epidemics. The study also suggests that future efforts to promote public health monitoring adherence should consider several key perceptions among people. These include self-confidence in performing the recommended behaviors and trust in program officials. It also is important for people to have the material tools they need to perform monitoring, for example, thermometers. Future initiatives should focus on bolstering such perceptions and ensuring such tools are available for participants.
